# Supplementary material for: High-pressure phase diagrams of FeSe1−xTex: correlation between suppressed nematicity and enhanced superconductivity
Source: Nat Commun. 2021 Jan 15;12:381. doi: 10.1038/s41467-020-20621-2 (PMC7810696; doi:10.1038/s41467-020-20621-2)
Supplement: Supplementary file 1 — Supplementary Information [file 41467_2020_20621_MOESM1_ESM.pdf]

Supplementary Information

High-pressure phase diagrams of $FeSe_{1-x}Te_x$: Correlation between suppressed nematicity and enhanced superconductivity

K. Mukasa,[1] K. Matsuura,[1] M. Qiu,[1] M. Saito,[1] Y. Sugimura,[1]
K. Ishida,[1] M. Otani,[2] Y. Onishi,[2] Y. Mizukami,[1,2] K. Hashimoto,[1,2]
J. Gouchi,[3] R. Kumai,[4] Y. Uwatoko,[3] and T. Shibauchi[1,2]

[1]*Department of Advanced Materials Science, University of Tokyo, Kashiwa, Chiba 277-8561, Japan*

[2]*Department of Applied Physics, University of Tokyo, Hongo, Tokyo 113-8656, Japan*

[3]*Institute for Solid State Physics, University of Tokyo, Kashiwa, Chiba, 277-8581, Japan*

[4]*Condensed Matter Research Center and Photon Factory, IMSS, KEK, Tsukuba, Ibaraki 305-0801, Japan*

Supplementary Fig. 1: Resistive determination of nematic transition temperature at ambient pressure.

Supplementary Fig. 2: Resistive determination of nematic and magnetic transition temperatures under pressure for x(Te) ≈ 0.04.

Supplementary Fig. 3: Resistive determination of nematic and magnetic transition temperatures under pressure for x(Te) ≈ 0.06.

Supplementary Fig. 4: Resistive determination of nematic and magnetic transition temperatures under pressure for x(Te) ≈ 0.10.

Supplementary Fig. 5: Resistive determination of nematic transition temperature under pressure for x(Te) ≈ 0.14.

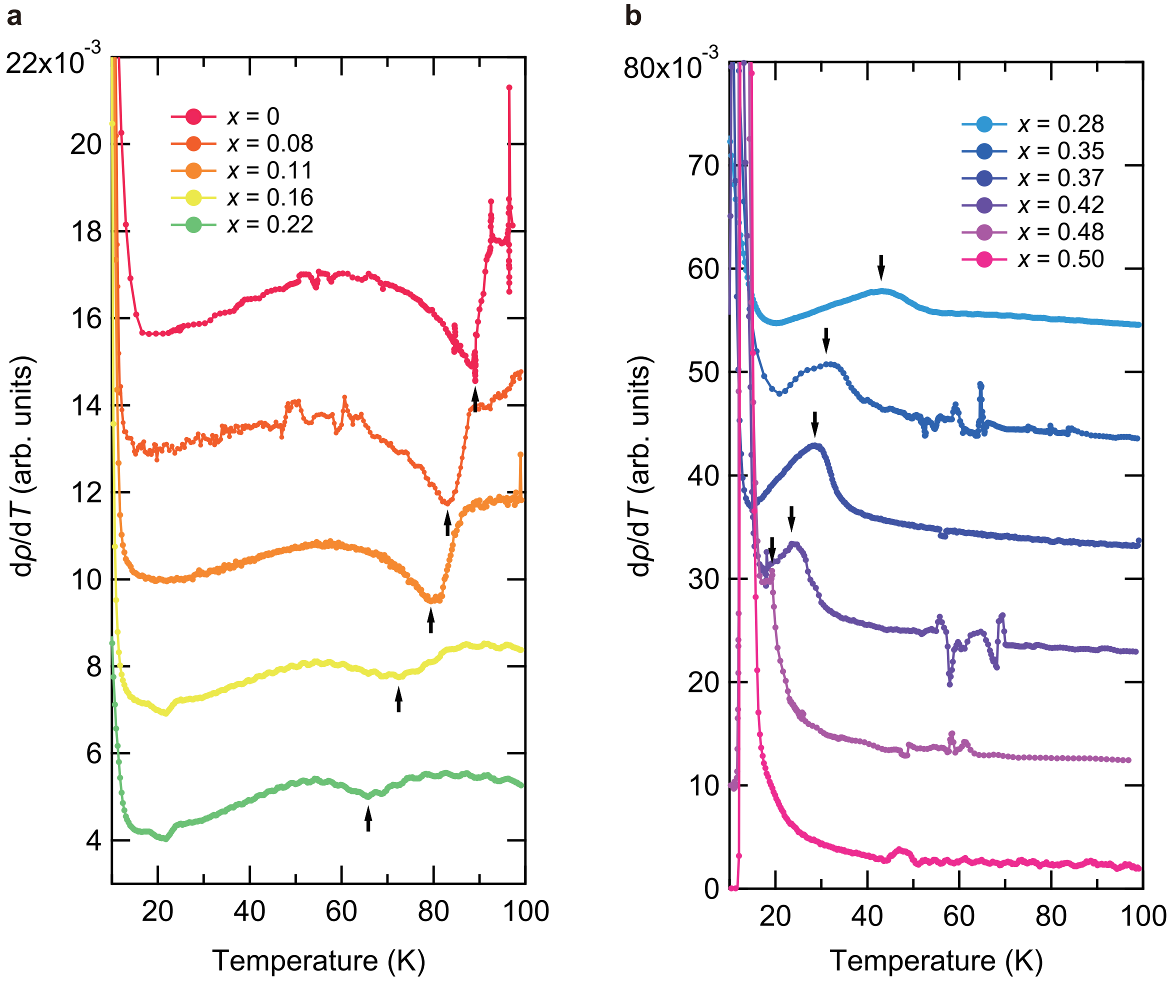

Supplementary Fig. 1. **Resistive determination of nematic transition temperature at ambient pressure. a**, Temperature dependence of $\mathrm{d}\rho/\mathrm{d}T$ for $0 \leq x(\mathrm{Te}) \leq 0.22$ in $\mathrm{FeSe}_{1-x}\mathrm{Te}_x$. **b**, Temperature dependence of $\mathrm{d}\rho/\mathrm{d}T$ for $0.28 \leq x(\mathrm{Te}) \leq 0.50$. Data are shifted vertically for clarity. The arrows indicate the nematic transition temperature T_s. At T_s the temperature dependence of resistivity $\rho(T)$ shows a kink anomaly with either upward or downward change depending on the condition. Thus we use dip or peak in the temperature derivative curves to define T_s.

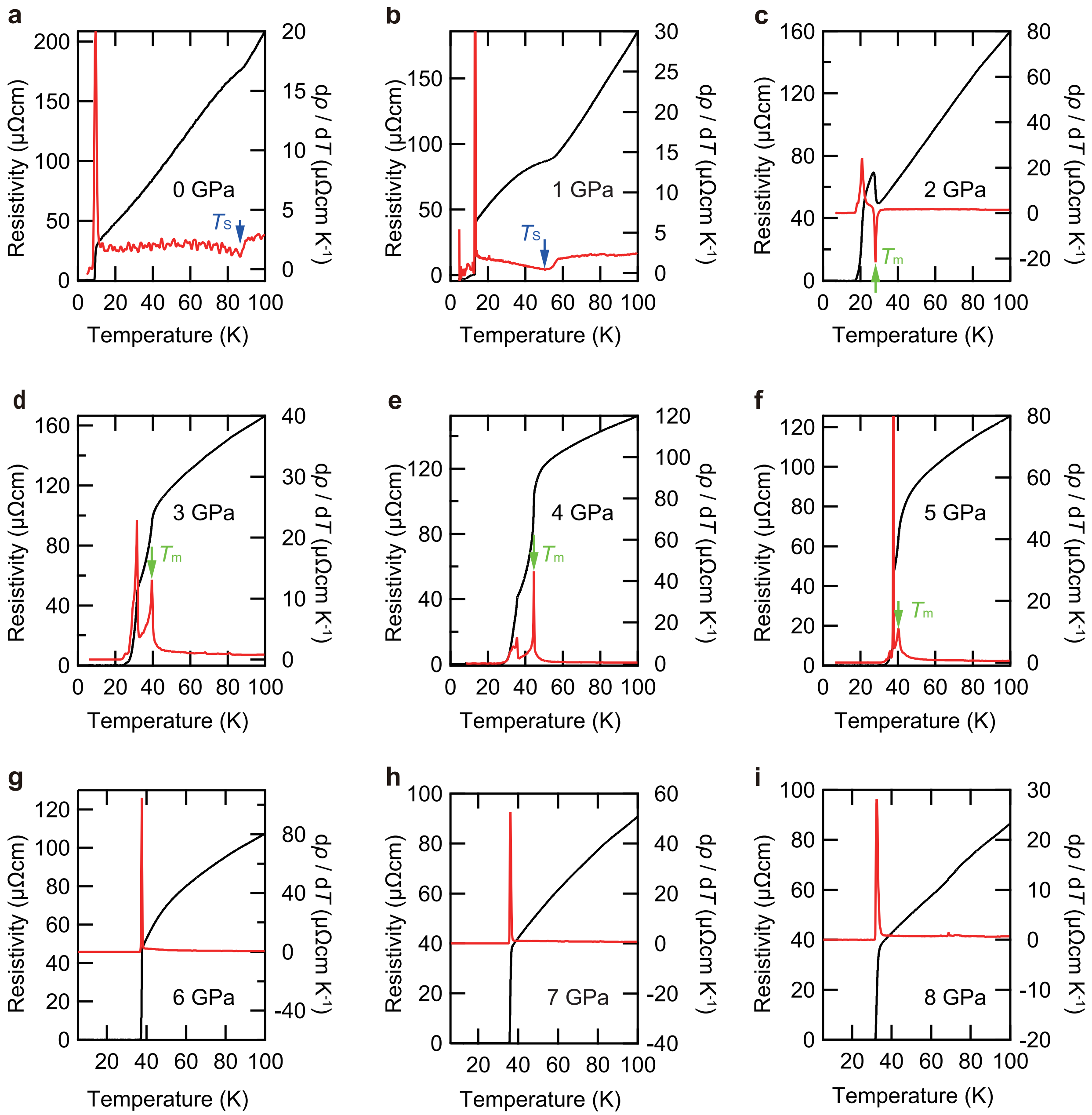

Supplementary Fig. 2. Resistive determination of nematic and magnetic transition temperatures under pressure for x(Te) $\approx$ 0.04. a-i, Temperature dependence of resistivity ρ (black, left axis) and $\mathrm{d}\rho/\mathrm{d}T$ (red, right axis) measured at 0-8 GPa with 1-GPa intervals. Arrows mark the nematic (T_s) and magnetic (T_m) transition temperatures.

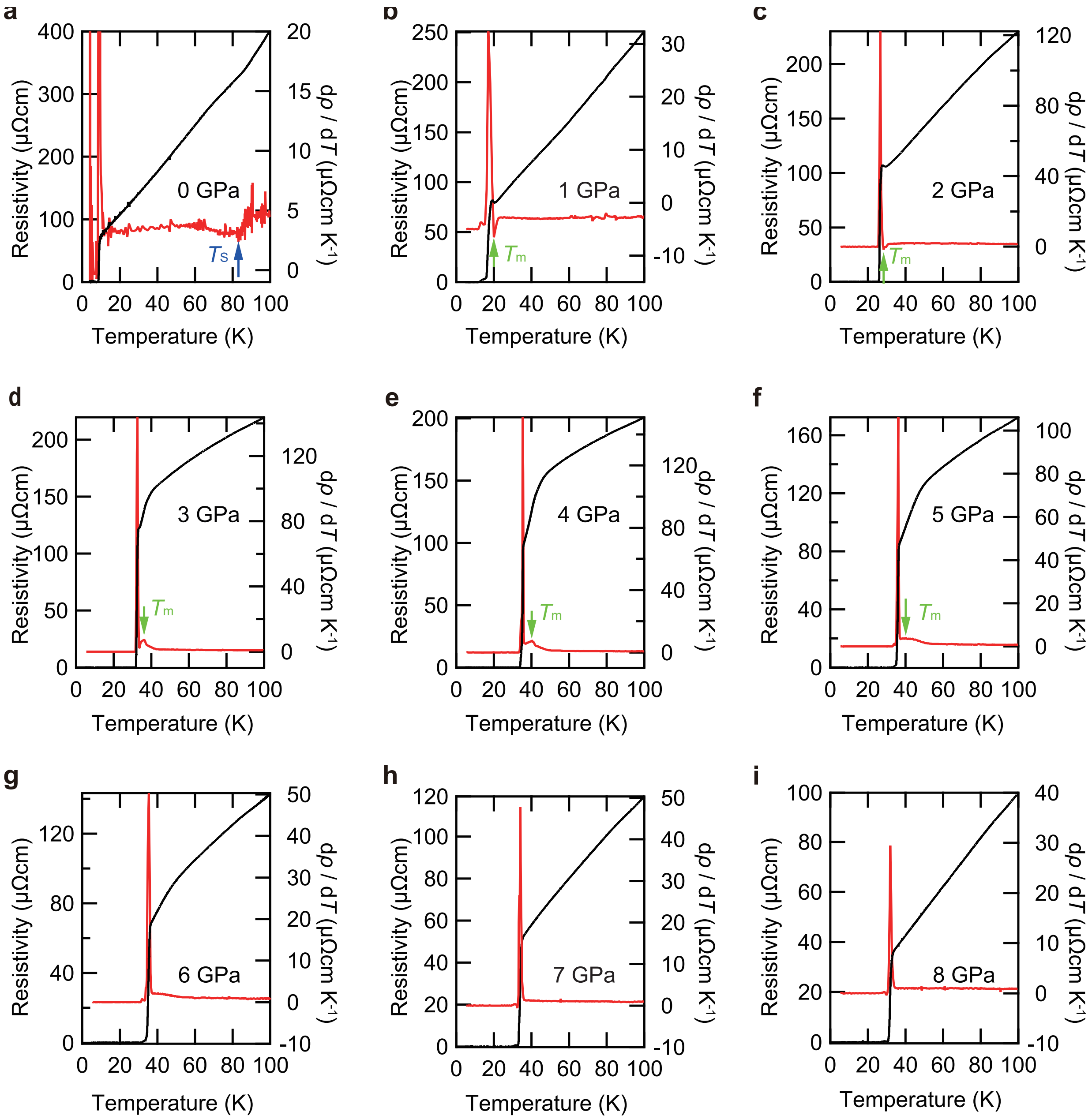

Supplementary Fig. 3. **Resistive determination of nematic and magnetic transition temperatures under pressure for x(Te) ≈ 0.06. a-i**, Temperature dependence of resistivity ρ (black, left axis) and $\mathrm{d}\rho/\mathrm{d}T$ (red, right axis) measured at 0-8 GPa with 1-GPa intervals. Arrows mark the nematic (T_s) and magnetic (T_m) transition temperatures.

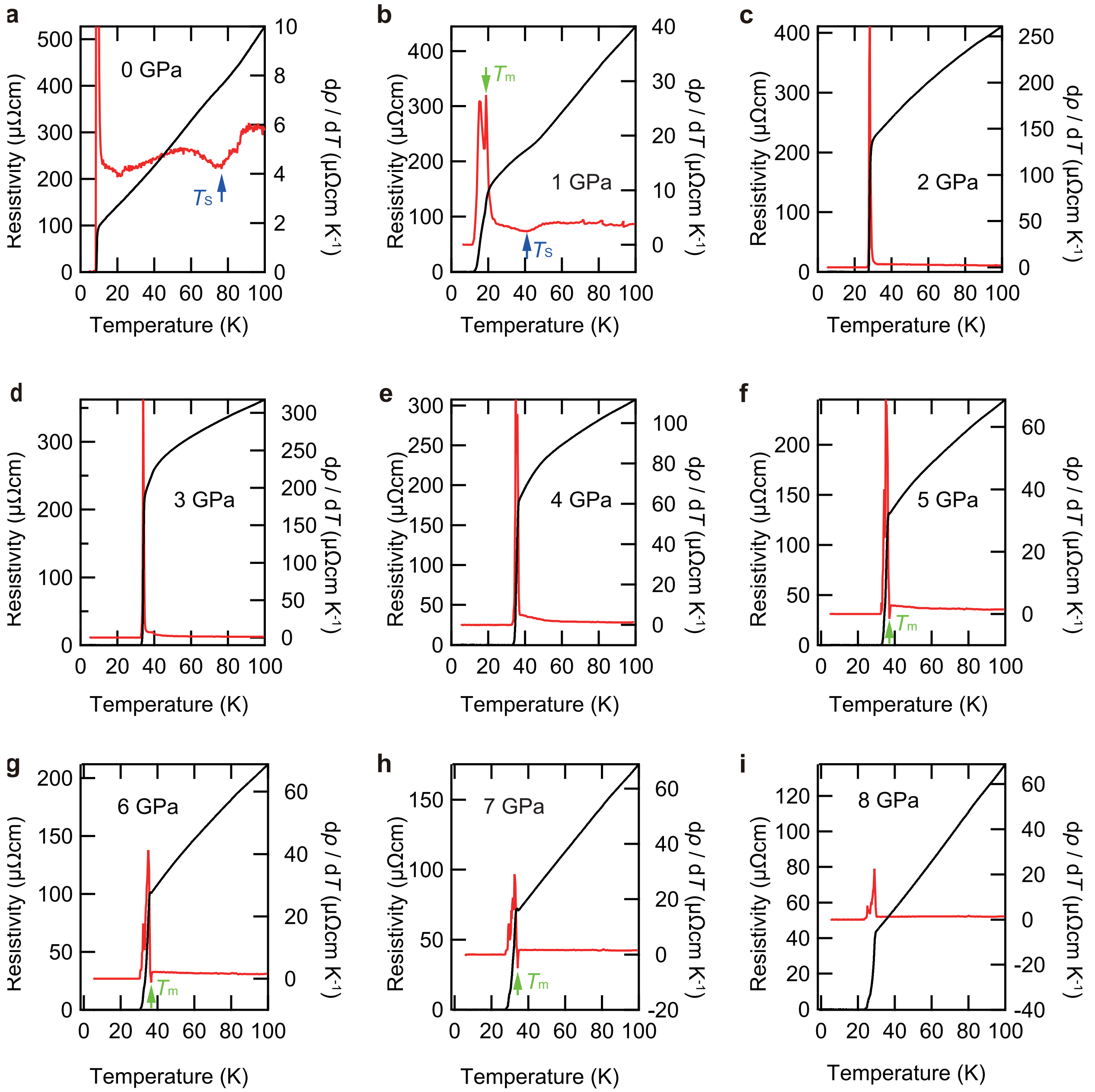

Supplementary Fig. 4. Resistive determination of nematic and magnetic transition temperatures under pressure for x(Te) ≈ 0.10. a-i, Temperature dependence of resistivity ρ (black, left axis) and $\mathrm{d}\rho/\mathrm{d}T$ (red, right axis) measured at 0-8 GPa with 1-GPa intervals. Arrows mark the nematic (T_s) and magnetic (T_m) transition temperatures.

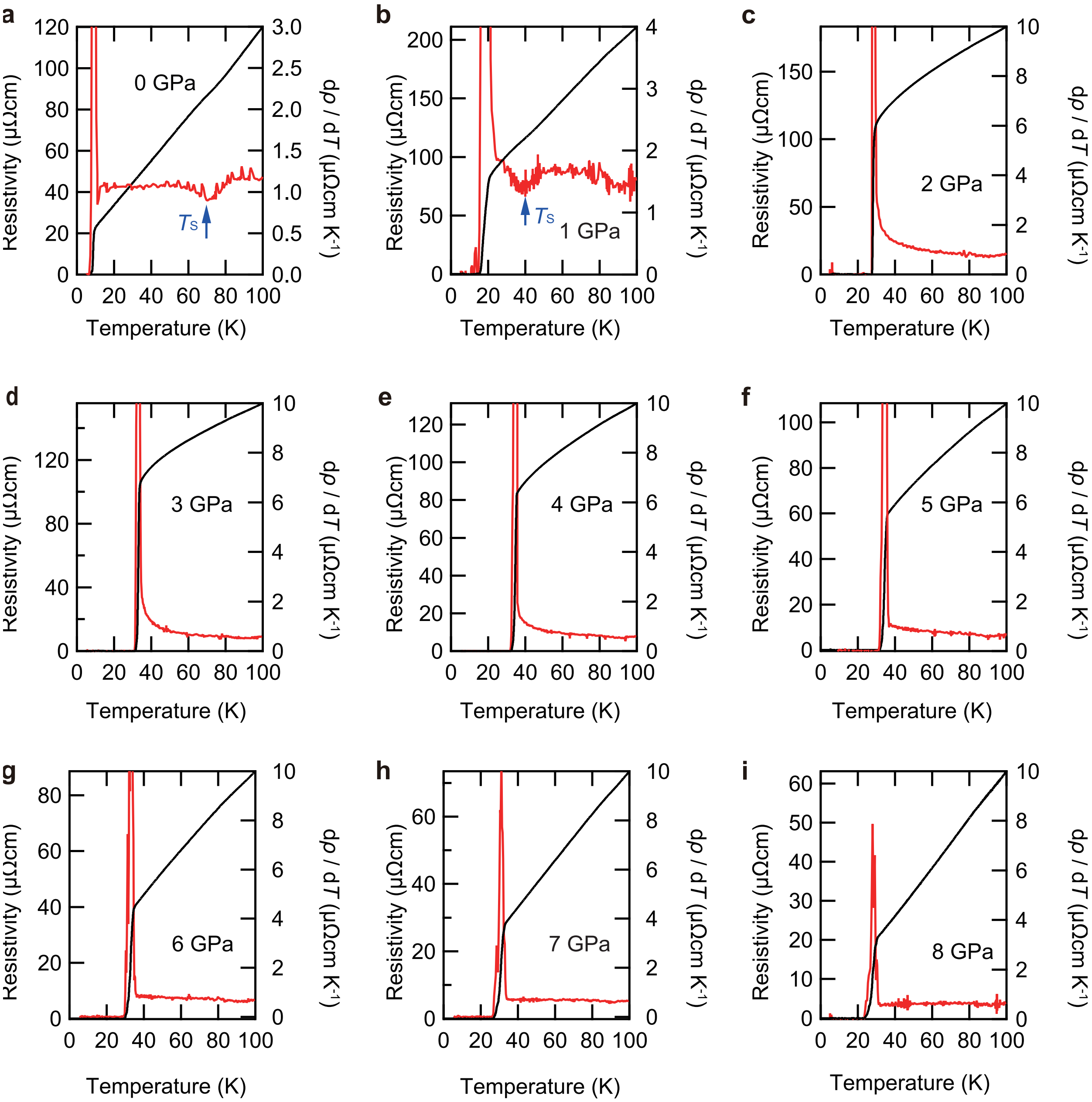

Supplementary Fig. 5. Resistive determination of nematic transition temperature under pressure for x(Te) ≈ 0.14. a-i, Temperature dependence of resistivity ρ (black, left axis) and $\mathrm{d}\rho/\mathrm{d}T$ (red, right axis) measured at 0-8 GPa with 1-GPa intervals. Arrows mark the nematic (T_{s}) transition temperatures.
